# Deglycosylation and label-free quantitative LC-MALDI MS applied to efficient serum biomarker discovery of lung cancer

**DOI:** 10.1186/1477-5956-9-18

**Published:** 2011-04-08

**Authors:** Atsuhiko Toyama, Hidewaki Nakagawa, Koichi Matsuda, Nobuhisa Ishikawa, Nobuoki Kohno, Yataro Daigo, Taka-Aki Sato, Yusuke Nakamura, Koji Ueda

**Affiliations:** 1 Graduate School of Frontier Sciences, The University of Tokyo, Kashiwanoha5-1-5, Chiba, Japan; 2 Laboratory for Biomarker Development, Center for Genomic Medicine, RIKEN, Tsurumiku-Suehirocho1-7-22, Yokohama, Japan; 3 Laboratory of Molecular Medicine, Human Genome Center, Institute of Medical Science, The University of Tokyo, Shirokanedai4-6-1, Tokyo, Japan; 4 Department of Molecular and Internal Medicine, Hiroshima University, Minamiku-Kasumi1-2-3, Hiroshima, Japan; 5 Shimadzu Corporation, Nishinokyo-Kuwabaracho1, Kyoto, Japan

## Abstract

**Background:**

Serum is an ideal source of biomarker discovery and proteomic profiling studies are continuously pursued on serum samples. However, serum is featured by high level of protein glycosylations that often cause ionization suppression and confound accurate quantification analysis by mass spectrometry. Here we investigated the effect of N-glycan and sialic acid removal from serum proteins on the performance of label-free quantification results.

**Results:**

Serum tryptic digests with or without deglycosylation treatment were analyzed by LC-MALDI MS and quantitatively compared on the Expressionist Refiner MS module. As a result, 345 out of 2,984 peaks (11.6%) showed the specific detection or the significantly improved intensities in deglycosylated serum samples (*P *< 0.01). We then applied this deglycosylation-based sample preparation to the identification of lung cancer biomarkers. In comparison between 10 healthy controls and 20 lung cancer patients, 40 peptides were identified to be differentially presented (*P *< 0.01). Their quantitative accuracies were further verified by multiple reaction monitoring. The result showed that deglycosylation was needed for the identification of some unique candidates, including previously unreported O-linked glycopeptide of complement component C9.

**Conclusions:**

We demonstrated here that sample deglycosylation improves the quantitative performance of shotgun proteomics, which can be effectively applied to any samples with high glycoprotein contents.

## Background

Since analyses of the serum proteome hold great promise for non-invasive detection of cancers and other diseases, various techniques for quantitative proteomic profiling have been developed to identify novel protein biomarkers[1,2]. These include labeling methods using stable isotopes such as ICAT (Isotope-coded affinity tags)[3], ^13^CNBS (2-nitrobenzenesulfenyl)[4], SILAC (Stable isotope labeling with amino acids in cell culture)[5] and iTRAQ (Isobaric tags for relative and absolute quantification)[6]. Control and test samples are labeled with reagents with different isotopic composition of ^12/13^C, ^14/15^N and/or ^16/18^O, and detected simultaneously by mass spectrometry so that the intensities of isotopically resolved peak-pairs (or peak groups) represent the quantitative ratio of control and test samples. Although the precision of quantification is very high (typically 10% relative standard deviation)[7] because of the identical separation and detection, isotopic labeling limits the number of samples to be directly compared, which makes it unsuitable for analysis of a large number of clinical samples needed for biomarker discoveries. In contrast, label-free quantification methods deal with independently-acquired mass spectrometry data from essentially unlimited number of samples. Quantification based on ion intensities (extracted ion chromatograms) is known to have at least three orders of linear dynamic range[8,9], and can potentially cover wide proteome in complex samples such as serum. It is advantageous that label-free systems do not involve sample mixing prior to detection because target proteins that are only presented in test samples are effectively diluted by mixing with control samples, rendering them more difficult to detect. Therefore, label-free quantification has emerged as an alternative approach for biomarker discovery, which requires sufficient sample sizes to overcome individual variability in clinical samples and technical bias in sample preparation and analysis batch[10].

High content of glycoproteins is another feature of serum that should be considered when performing quantitative proteomic analysis. Recent advances in glycoproteomic analysis using mass spectrometry have made it possible to exhaustively identify N-linked glycopeptides and their glycosylation sites[11,12]. These techniques involve enrichment of glycopeptides followed by enzymatic cleavage of N-glycans in order for efficient mass spectrometric analysis. Deglycosylation can be coupled with the incorporation of ^18^O stable isotope resulting in +3 Da mass shift of asparagine residues, which allows deterministic identification of glycosylation sites[13]. As these studies indicated, most of serum proteins are heavily glycosylated, however, potential effect of glycopeptides on ionization suppression of co-existing peptides had been overlooked. Glycopeptides carry large, hydrophilic carbohydrate moieties, which can cause substantial ionization suppression[14], hampering precise quantification particularly at low-concentration range.

To elucidate the extent to which the ionization of peptides is interfered by glycopeptides, the first part of this study describes the changes caused by serum deglycosylation in the MS peak profiles obtained by label-free shotgun proteomic analysis. Having shown the utility of deglycosylation, we next applied this principle to the biomarker screening of lung cancer. Because of the vast number of incidence and high mortality rate, lung cancer is considered to be one of the highest priorities for biomarker development. Using the serum samples of 10 healthy control, 10 early-stage (Stage I-II) cases and 10 advanced stage (Stages IIIb-IV) cases, we conducted a study that uniquely combined sample deglycosylation, label-free MALDI and multiple reaction monitoring (MRM) mass spectrometry[15] and verified the result by western blotting. Taken together, we show herein that enzymatic removal of carbohydrate moieties results in recognizable improvement in the data quality of shotgun proteomics in terms of sensitivity and reproducibility, which should facilitate quantitative analysis of glycoprotein-rich samples.

## Results

### Quantitative MS analysis of deglycosylated serum

**Figure 1:**
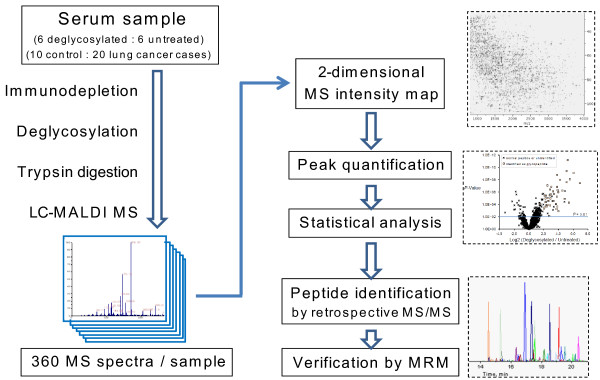
**Schematic of the LC-MALDI label-free quantification with deglycosylation**.

**Figure 2:**
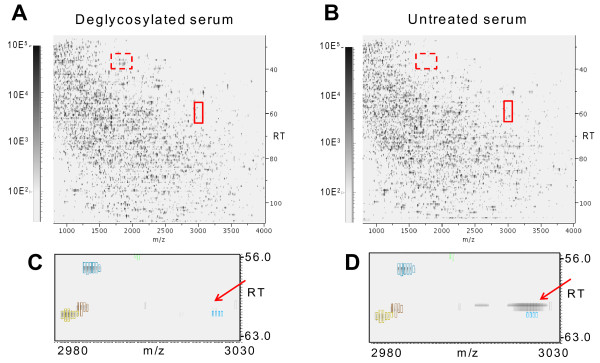
**Representative 2-dimesional signal intensity maps visualizing the peptide profiles of (A) tryptic digest of deglycosylated serum, and (B) untreated serum, within the range of m/z 800-4000, and 25-110 minutes of retention time**. An example of obvious peak appearance in deglycosylated serum is indicated by broken boxes. (C) and (D) display the expanded views of the closed box in deglycosylated sample and untreated sample, respectively. The rectangles outlining the MS signal represent peak detection and their color coordination represents peak clustering.

**Figure 3:**
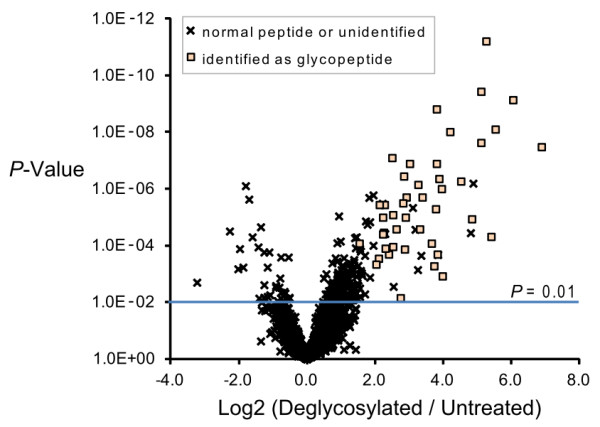
**Volcano plot showing the difference in the peptide profiles of deglycosylated and untreated serum sample**. Crosses represent peak clusters identified as normal peptides or not identified in the analysis, and squares represent deglycosylated peptides identified with +3 Da modification.

In order to examine the effect of deglycosylation on data content and quality, tryptic digest of serum proteins was prepared with or without the removal of N-glycans and sialic acids (n = 6), and analyzed on the LC-MALDI label-free quantification platform as summarized in Figure 1. Figure 2 shows the 2-dimensional MS signal intensity maps after data processing by Expressionist Refiner MS (Genedata). In total, 27,357 single peaks were detected, comprising 4,444 groups of peaks each representing unique peptide species (termed "peak clusters"). Examples of the differences in the peak profile between deglycosylated and untreated samples are illustrated in the 2D map. The broken box in Figure 2a shows emergence of prominent peaks, and the expanded views in Figure 2c and 2d (arrows) illustrate the loss of intact glycopeptide peaks. The peak clusters subjected to comparative analysis were selected by eliminating peaks that were not presented in all of the 6 replicate runs. This filtering was performed separately for deglycosylated and untreated samples, yielding 2,984 and 2,610 peak clusters, respectively. Deglycosylation thus resulted in 14.3% increase in the number of reproducibly detectable peaks. The signal intensities of these peak profiles were then directly compared by t-test as summarized in the volcano plot (Figure 3). 221 peak clusters displayed altered intensities, of which 188 were higher in deglycosylated samples and 33 peak clusters vice versa (*P *< 0.01). Present/absent search revealed that 157 peak clusters were found specifically in deglycosylated samples (Figure S-1, Additional File 1). Combined, 345 of 2,984 peak clusters (11.6%) detected in deglycosylated samples were enhanced by deglycosylation, as opposed to 33 of 2,610 peak clusters (1.3%) in untreated serum.

The composition of the peak profiles was analyzed by exhaustive MS/MS analysis, resulting in 1,735 peptide identifications, including 153 originally-glycosylated peptides that were identified with ^18^O-incorporated N-glycosylation sites (Table S-2, Additional File 1). By matching the peptide IDs to the statistical analysis, we found that 97 of 345 peak clusters overrepresented in deglycosylated serum were originally-glycosylated peptides. These peak clusters emerged as the product of deglycosylation and accounted for most of the major fold-changes in the volcano plot as indicated with squares in Figure 3. The remaining 248 peak clusters, as well as the 33 peak clusters that diminished by deglycosylation, were non-glycopeptides. There were no apparent features common to these peptides. Rather, signal intensities were affected by the local peptide composition, such that alleviation of suppressive analytes led to signal enhancement and emergence of competing analytes led to diminished signal. The result shown here, that the number of enhanced peaks was by far greater than those that diminished, suggest that the ionization suppression effect exerted by intact glycopeptides was more extensive than the deglycosylated counterpart.

**Figure 4:**
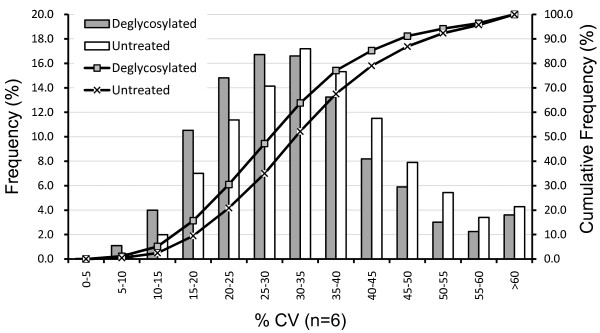
**A frequency distribution plot of the coefficients of variations (CV) calculated for each of the 2,356 common peak clusters**. Bars represent the frequency percentage and lines represent the cumulative frequency. Filled bars and boxes, replicates with deglycosylation; blank bars and crosses, untreated replicates (n = 6).

To investigate whether deglycosylation had any effect on the quality of quantitative data, reproducibility of six replicating runs was evaluated by calculating the correlation coefficients of the intensities of 2,356 clusters that were detected in all 12 runs. The correlation coefficients were calculated for all 15 possible combinations of 6 individual replicates within the experimental group. They ranged from 0.895 to 0.916 with mean value 0.899 (n = 15) in the untreated group and from 0.898 to 0.944 with mean value 0.914 (n = 15) in the deglycosylated group, suggesting that deglycosylation generally results in more reproducible data than the untreated (*P *< 0.002, t-test). Furthermore, coefficients of variation (CV) of the signal intensities for the same 2,356 peak clusters were calculated and compared between the two experimental groups (Figure 4). The frequency distribution of CV showed that deglycosylationtreatement resulted in slightly lowering the median CV from 34% to 31%. It is clear from these data that the effect of deglycosylation well extends to non-glycosylated peptides and enhances sensitivity and reproducibility.

### Lung cancer biomarker screening

Deglycosylation-LC-MALDI platform was applied to the profiling of serum proteins for lung cancer biomarker screening. Control and lung cancer sera were immunodepleted, deglycosylated, purified on SDS-PAGE, in-gel digested and analyzed by LC-MALDI in the same way as described above. 30 mass chromatograms were processed simultaneously on Expressionist Refiner MS, and 23,453 peaks were detected, which were grouped into 6,186 peak clusters. Then we compared the two experimental groups, control (n = 10) and lung cancer (n = 20) by t-test and identified 63 peak clusters showing P < 0.01. In addition, 13 peak clusters were selected that have valid value of less than or equal to 2 in one experimental group and greater than 50% valid value proportion in the other. The total of 76 clusters was manually inspected and retrospective MS/MS was acquired by selecting the highest-expressing sample and the fraction spot of maximum elution for optimum MS/MS acquisition efficiency. As a result, 25 candidate proteins, comprising 40 candidate peptides, were identified (Table 1).

**Table 1:** List of lung cancer biomarker candidates screened by label-free MALDI MS and their verification result on MRM.

			MALDI MS		MRM		
					
Uniprot ID	Protein Name	Peptide Sequence	t-test ^†^	Log2(LC/Control)	t-test ^‡^	Log2(LC/Control)	Correlation Coefficient
P04217	Alpha-1B-glycoprotein	CEGPIPDVTFELLREGETKAVK	-	0.67	-	-	-
		FALVREDR	3.8E-03	-0.38	0.13	0.24	0.315

P01011	Alpha-1-antichymotrypsin	EQLSLLDRFTEDAK	1.4E-03	0.80	0.077	0.33	0.106
		FTEDAKRLYGSEAFATDFQDSAAAK	4.6E-03	0.58	-	-	-
		PQDTHQSR	5.6E-03	0.84	-	-	-

P43652	Afamin	DGLKYHYLIR	1.1E-03	-0.93	-	-	-

P02746	Complement C1q subcomponent subunit B	GNLCVNLMR	6.5E-03	0.87	-	-	-

P00736	Complement C1r subcomponent	CLPVCGKPVNPVEQR	-	0.42	-	-	-
		DYFIATCK	6.9E-03	0.49	0.15	0.14	-0.238

P00450	Ceruloplasmin	YTVNQCR	0.017	1.1	3.1E-04	0.29	0.411*

P10909	Clusterin	YVNKEIQNAVNGVK	0.01	0.33	0.19	-0.87	-0.015

P06681	Complement C2	TAVDHIREILNINQK	2.7E-03	1.4	0.051	0.23	N/A

P01024	Complement C3	AGDFLEANYMNLQR	5.9E-05	-1.4	3.8E-04	-1.3	0.690**
		ILLQGTPVAQMTEDAVDAER	2.1E-04	-1.3	0.019	-0.87	0.448*
		KGYTQQLAFR	2.8E-03	-1.1	3.9E-05	-1.3	0.800**
		QPSSAFAAFVKR	6.7E-03	-1.3	2.0E-03	0.24	-0.455
		WLNEQR	2.5E-03	-1.0	7.8E-06	-1.5	0.521**

P01031	Complement C5	FWKDNLQHKDSSVPNTGTAR	0.012	0.67	-	-	-
		TLRVVPEGVKR	0.066	0.80	-	-	-

P13671	Complement component C6	IEEADCKNKFR	0.011	1.2	-	-	-

P10643	Complement component C7	VFSGDGKDFYR	9.5E-03	0.81	-	-	-

P02748	Complement Component C9	FTPTETNKAEQCCEETASSISLHGK	2.4E-04	1.4	6.1E-03	0.49	0.472*
		QYTgTSYDPELTESSGSASHIDCR	1.9E-03	1.1	3.8E-03	0.57	0.769**

P22792	Carboxypeptidase N subunit 2	SQCTYSNPEGTVVLACDQAQCR	1.4E-03	0.56	0.062	0.16	0.372

P00748	Coagulation factor XII	CTHKGRPGPQPWCATTPNFDQDQR	4.3E-03	1.2	-	-	-

Q9UGM5	Fetuin-B	MSPPQLALNPSALLSR	3.0E-03	0.62	0.03	0.58	0.189

P02751	Fibronectin	AQITGYR	1.1E-03	-0.47	0.077	-0.18	0.774**
		GFNCESKPEAEETCFDKYTGNTYR	1.9E-03	-0.69	0.42	-0.11	0.146
		IGFKLGVRPSQGGEAPR	5.7E-03	-0.90	-	-	-

P26927	Hepatocyte growth factor-like protein	RVDRLDQR	6.1E-03	-0.57	0.013	0.38	-0.128

P18065	Insulin-like growth factor-binding protein 2	LAACGPPPVAPPAAVAAVAGGAR	2.5E-03	0.67	-	-	-

Q06033	Inter-alpha-trypsin inhibitor heavy chain H3	EHLVQATPENLQEAR	4.6E-03	0.97	3.6E-03	0.65	0.578**

Q14624	Inter-alpha-trypsin inhibitor heavy chain H4	EKNGIDIYSLTVDSR	4.7E-04	0.65	0.062	0.40	0.368
		ETLFSVMPGLK	-	0.09	0.01	0.42	0.331
		MNFRPGVLSSR	9.8E-03	0.40	1.0E-03	0.63	0.413*
		SPEQQETVLDGNLIIRYDVDR	0.013	1.2	-	-	-

P03952	Plasma kallikrein	CQFFTYSLLPEDCKEEKCK	-	-0.32	-	-	-

P01042	Kininogen-1	RPPGFSPF	2.0E-04	-2.0	5.1E-04	-1.74	0.876**

P27918	Properdin	TCNHPVPQHGGPFCAGDATR	7.2E-04	-0.52	-	-	-

Q13103	Secreted phosphoprotein 24	DSGEDPATCAFQR	3.7E-03	-0.53	-	-	-

^†^: "-" indicates candidate screened by present/absent search,

‡: "-" indicates no MRM data obtained, *: *P *< 0.05, **: *P *< 0.01,

g: indicates site of O-linked glycosylation

N/A: valid value too small for calculation of correlation threshold.

**Figure 5:**
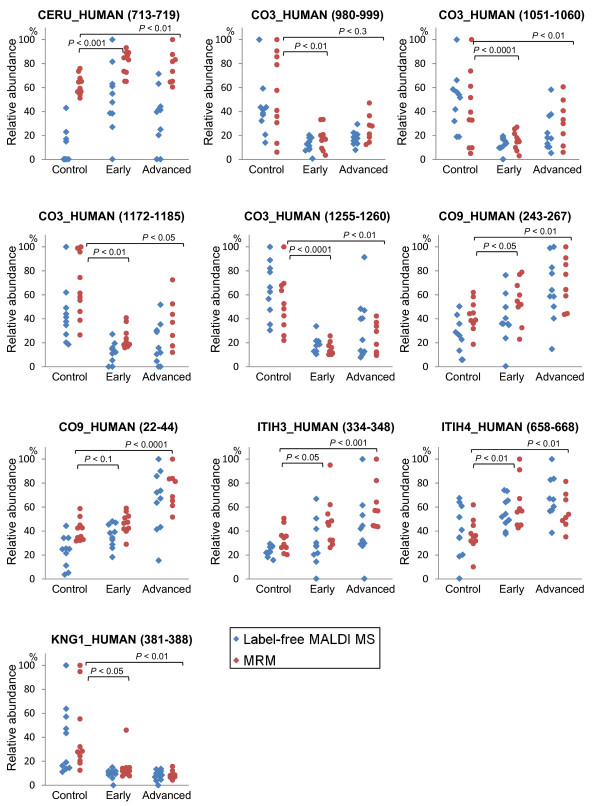
**Dot plots showing the relative quantifications in 10 controls, 10 early stage and 10 advanced stage lung cancer cases of the 10 candidate peptides, subtitled with SwissProt IDs and amino acid numbers in parenthesis**. Diamond dots aligned to the left represent quantification by MALDI MS, and circular dots aligned to the right by MRM, normalized and plotted on the same scale. The *P*-values presented were calculated using the results of MRM.

Next, these candidates were verified at peptide level by the MRM-based relative quantification analysis using the same preparation batch of serum tryptic digest as used for MALDI MS analysis as it is previously reported that LC-MALDI measurement is the most significant source of technical variability above sample preparation[16] (see Table S-3, Additional File 1 for the list of MRM transitions). This strategy was aimed at eliminating false-positive results from the long list of candidates and facilitating the selection of appropriate target for validation study. Of the 23 peptides that we found working MRM transitions, 11 peptides showed significant correlation (*P *< 0.05 by Pearson's correlation coefficient) in results obtained by MRM and MALDI MS, and 10 peptides (6 proteins) fulfilled *P *< 0.05 in both analyses. These peptide candidates were derived from ceruloplasmin, complement C3, complement component C9, inter-alpha-trypsin inhibitor heavy chain H3, inter-alpha-trypsin inhibitor heavy chain H4 and kininogen-1. The quantification results were summarized as dot-plots in Figure 5 to illustrate the potential performance of the 10 peptides as biomarker candidates. Raw data was normalized to the total detection and the samples were grouped as normal control, early stage (Stage I/II) and advanced stage (stage IIIb/IV) lung cancer cases. As the *P*-values indicate, most of the candidates showed significant response to cancer state even at early stages.

**Figure 6:**
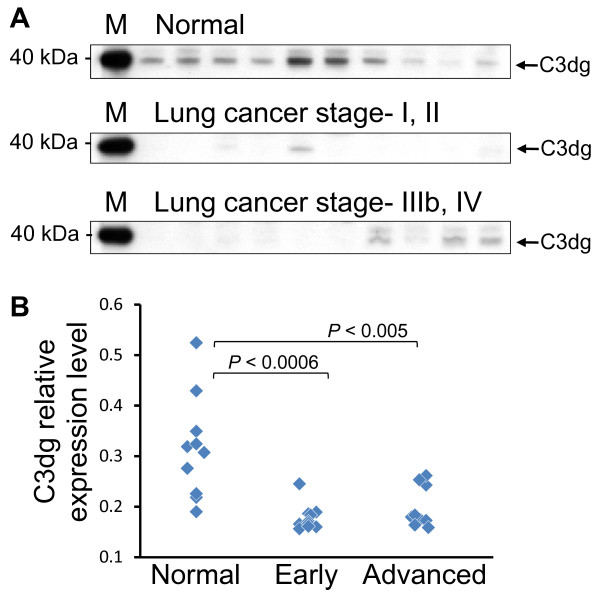
**Immunoblot verification of complement C3dg serum levels in the study subjects**. (A) Detection of 39 kDa C3dg fragment from 0.02 μL of crude serum using anti-C3 antibody. Lanes represent 10 subjects each of normal control, early stage lung cancer (stage-I/II) and advanced stage lung cancer (stage-IIIb/IV). (B) Dot plot of the data shown in (A), quantified as the ratio of C3dg detection level to the normalization lane M, a 40 kDa size marker that has cross-reactivity with the secondary antibody.

Further verification was performed by western blotting of complement C3 protein. Interestingly, expression of 39 kDa subunit of C3 protein was strongly suppressed in early-stage patients (Figure 6A). Since this subunit was also detected by the monoclonal antibody raised against C3d fragment (Figure S-2 Additional File 1), the 39 kDa subunit was assigned to be C3dg fragment[17]. This fragment encompasses all of the four C3 peptide candidates identified by LC-MALDI screening, and semi-quantitative analysis of the immunoblot (Figure 6B) almost exactly reproduced the screening result. Moreover, C3dg fragment was shown to escape immunodepletion by MARS-Hu14 column (Figure S-2). Therefore, the apparent difference in complement C3 abundance observed in the screening was reflecting the degree of proteolytic degradation associated with lung cancer.

**Figure 7:**
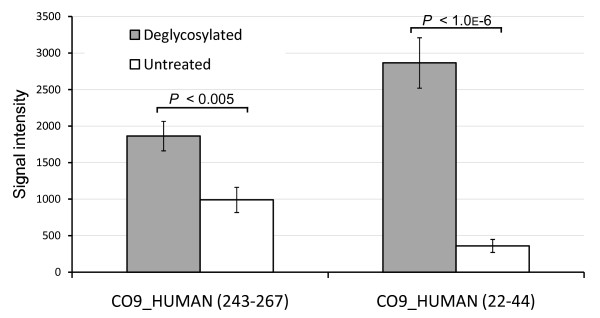
**Bar chart representing the signal intensities of complement component C9 peptides detected from the tryptic digest of deglycosylated serum (filled bars) and untreated serum (open bars), n = 6**. The numbers in parenthesis are amino acid numbers: 243-267 corresponds to _243_FTPTETNKAEQCCEETASSISLHG_267_K and 22-44 corresponds to _22 _QYTgTSYDPELTESSGSASHIDC_44_R, where "g" represents the site of O-linked glycan attachment. Error bars are one standard deviation.

Finally, the benefit of deglycosylation in this biomarker screening was assessed by mining the candidate peptides from the control experiment (comparing deglycosylated and untreated serum) and verifying whether or not deglycosylation facilitated biomarker identification. Figure 7 shows the levels of complement component C9 peptides in the control experiment, which were clearly overrepresented by deglycosylation. Notably, the signal intensity of non-glycopeptide (243-267) was doubled, reiterating the observation that non-glycopeptide was also subject of signal enhancement by deglycosylation. Moreover, the signal intensity of peptide (22-44) was increased by 10-fold. Since this peptide was identified as O-linked glycopeptide (attachment of N-acetylgalactosamine and galactose as predicted from the m/z shift), the addition of sialidase probably contributed to reduction of glycan complexity and increased ionization efficiency. The sialylated counterpart was not detected in the untreated control.

## Discussion

The aim of this study was to introduce deglycosylation as a facile and universal sample preparation step in shotgun proteomic analysis because we expected that the undermining effect exerted by the large proportion of glycopeptides was more extensive than previously considered. Therefore, for the first time, we have performed a direct comparison between deglycosylated and untreated serum samples, and showed that deglycosylation actually results in improvement of the shotgun peak profile. This was observed in terms of both acquisition of unique peaks and enhancement of existing peaks.

The acquisition of unique peaks by deglycosylation was expected, as it is widely recognized that deglycosylated peptides have much higher ionization efficiency than the corresponding glycopeptides[18,19]. It is well established that deglycosylation results in the detection of "new" peaks and increases the depth of information acquired by shotgun analysis[20,21]. However, this study revisited the same phenomenon from different perspective. Our novel finding was that signal enhancement by deglycosylation extended up to 8% of existing non-glycopeptide peaks (Figure 3). This result strongly suggested that there was notable ionization suppression effect exerted by glycopeptides on co-existing analytes, and that signal intensities were enhanced through alleviation of suppression in deglycosylated sample. Incorporation of ^18^O into the site of deglycosylation by H_2_^18^O was utilized to facilitate the identification of glycopeptides. The potential pitfalls of this approach as pointed out by Angel et al.[22] was circumvented by performing deglycosylation before tryptic digestion, and our data showed that 73% of identified glycopeptides fulfilled the biological consensus NxT/S. This strategy helped to confirm that only a small proportion of peaks that were enhanced by deglycosylation were actually glycosylated. We further demonstrated the evidence that deglycosylation improves the reproducibility of replicate measurements to some extent (Figure 4). The level of technical variability, median CV of 31%, was comparable to previously reported label-free quantification methods based on LC-ESI-MS[8] or LC-MALDI MS[16].

Unfortunately, previous studies on ionization suppression effects had mainly focused on selective detection of glycopeptides in mixtures of peptides[23], and cannot explain the phenomenon addressed here. Separate investigation needs to be conducted in order to elucidate the mechanism and the extent to which glycopeptides interfere with the ionization of co-existing peptides.

Serum deglycosylation coupled with label-free quantification was applied to biomarker screening for lung cancer and led to the identification of unique biomarker candidates including the fragmentation state of complement C3, complement component C9 peptide with novel O-linked carbohydrate and Kininogen-1 peptide with C-terminal Phe[24]. The benefit of deglycosylation in the biomarker screening was demonstrated by enhanced detectability of the complement component C9 peptides, particularly for O-linked glycopeptide whose intact form with sialic acid was hardly detected. Since many O-linked glycans contain sialic acid, the data presented here demonstrates a high potential of sialidase usage for comprehensive analysis of O-linked glycans.

As with other label-free quantitative proteomics, more proteins were quantified by single peptide than those quantified by multiple peptides[25] due to the high technical variability associated with label-free shotgun analysis. Therefore, we employed MRM to complement the screening by MALDI MS with the aim of eliminating false-positive results. This approach successfully ruled out many candidates while retaining confident candidates, as verified by western blotting experiment of complement C3.

Complement C3 is the major component of the classical complement pathway. Upon antigenic stimulation, C3 convertase cleaves C3 into C3a and C3b, which subsequently triggers reaction cascade leading to the formation of membrane attack complex, by either the classical or alternative pathway[17]. C3a contains a multiply interacting motif known as anaphylatoxin[26]. Recently, a number of proteomic studies have identified C3a as biomarker candidates for colon cancer[27], chronic hepatitis C and related hepatocellular carcinoma[28], insulin resistance/type-2 diabetes[29] and chronic lymphoid malignancies[30]. While upregulation of C3a is widely reported and interpreted as an indicator of primary inflammatory response, there is limited association reported between C3dg and cancer. C3dg is known as the ligand of complement receptor 2[31] and may be critically involved in cancer recognition. The mechanism by which the production of C3dg is suppressed in response to the onset of lung cancer requires further investigation.

In the Expressionist label-free quantification platform employed here, peptide peak clusters are defined by retention time-m/z coordinate on the 2D-map, enabling quantitative analysis without MS/MS information that were essential in other platforms[32-35]. The feature that provides the ground for this concept is perfect alignment of mass chromatograms in the retention time dimension because slight drift is unavoidable even in well-optimized separation system. In this respect, Expressionist demonstrated spectacular computational strength. The range of retention time drift in the 30 LC-MALDI analyses performed in this study was from -5 minutes to +5 minutes, a maximum of 10 minutes deviation, but the software was still capable of good alignment without any obvious retention time mismatch (data not shown). Therefore, variation in experimental conditions, such as changing the analytical column lot, should easily be tolerated. This feature enables integration of several, even retrospective, analyses, which is needed for the continuous pursuit for biomarker identification and validation.

Moreover, being a server-based module, Expressionist has greater data processing capability than other stand-alone software. This is another advantageous feature because, in general, attempts to increase proteome coverage involve vast increase in data amount, whether it be a multi-dimensional fractionation strategy[36,37] or an extremely long gradient separation[38]. Importantly, considering the fact that low-abundance analytes are more prone to ionization suppression[39], we speculate that the benefits of sample deglycosylation we addressed here would take greater effect with increasing dynamic range of detection. Such in-depth label-free analysis is currently not available, however, we demonstrated herein that current technology is already capable of large-scale label-free analysis, and we addressed its potentiality as a biomarker discovery platform. Taken together, we believe that sample deglycosylation will prove to be a valuable sample preparation protocol in shotgun proteomic analysis in near future for analyzing glycoprotein-rich samples.

## Conclusions

The studies described herein demonstrated that serum deglycosylation has positive effect on both data content and reproducibility through production of deglycosylated peptides and possibly through alleviation of ionization suppression by intact glycopeptides. The results therefore suggested the role of deglycosylation as a simple, indispensible method to improve the general performance of label-free quantification. Its first application to serum proteomic profiling by label-free LC-MALDI MS demonstrated that this strategy could lead to the identification of unique candidates, which could be effectively applied to any samples with high glycoprotein contents, such as other clinical body fluids, membrane proteomics or secretome analysis.

## Methods

### Reagents

Trizma base pH 8.3, iodoacetamide, ammonium bicarbonate, ammonium citrate, formic acid were purchased from Sigma (Saint Louis, MO). PlusOne grade SDS and dithiothreitol (DTT) were purchased from GE Healthcare (Uppsala, Sweden). CHAPS was purchased from Chemical Dojin (Kumamoto, Japan). N-glycosidase F was purchased from Roche (Basel, Switzerland). α2-3,6,8,9-neuraminidase was purchased from Merck (Darmstadt, Germany). Trypsin Gold was purchased from Promega (Madison, WI). H_2_^18^O was supplied from Cambridge Isotope Laboratories Inc. (Andover, MA). Alpha-cyano-4-hydroxycinnamic acid (CHCA) was purchased from Shimadzu-GLC (Kyoto, Japan). LC/MS grade acetonitrile and 25% trifluoroacetic acid (TFA) were purchased from Wako Pure Chemicals (Osaka, Japan).

### Serum Samples

Archived human serum samples were obtained with informed consent from 20 patients with lung adenocarcinoma and at Hiroshima University Hospital. Serum samples as normal controls were also obtained with informed consent from 13 healthy volunteers who received medical checkup at Hiroshima University Hospital (Table S-1, Additional File 1). Serum was collected using standard protocol from whole blood by centrifugation at 1500 × g for 10 min and stored at -150°C. This study was approved by individual institutional ethical committees.

### Immunodepletion

20 μL serum aliquots obtained from healthy volunteer were subjected to Multiple Affinity Removal System (Hu-14, 4.6 mm × 100 mm, Agilent Technologies, Santa Clara, CA) according to the manufacturer's protocol using a conventional HPLC system (Shimadzu Corp., Kyoto, Japan). The flow-through fraction was desalted with a protein separation column (mRP-C18, 4.6 mm × 50 mm, Agilent Technologies). Desalted serum proteins were dried with a SpeedVac evaporator.

### Deglycosylation

All solutions in the following deglycosylation step were freshly prepared with H_2_^18^O. Protein aliquots were dissolved in 12.5 μL of 2% SDS, 20 mM DTT, 20 mM Trizma-base pH 8.3 and heated to 100°C for 5 minutes. After cooling, 25 μL of 10% CHAPS, 83.4 μL 20 mM Trizma-base pH 8.3, 1 μL N-glycosidase F and 0.6 μL α2-3,6,8,9-neuraminidase were added in the written order with thorough mixing. H_2_^18^O was added in place of the enzymes for "untreated" samples. The reaction mixture was incubated at 37°C overnight.

### Tryptic digestion

Deglycosylated proteins were reduced by 10 mM DTT and incubated at 56°C for 15 minutes, followed by alkylation by 50 mM iodoacetamide at ambient temperature for 45 minutes in dark. 20% of the total reaction mixture was purified by SDS-PAGE, applying voltage until all of the proteins had entered the separating gel. Whole lanes were cut out and subjected to in-gel tryptic digestion. Briefly, gel slices were cut into small pieces and were washed 3 times in 30% acetonitrile 50 mM ammonium bicarbonate before digesting with 200 ng of trypsin in 100 μL 50 mM ammonium bicarbonate at 37°C overnight. Peptides were extracted by 2 rounds of 50% acetonitrile and 100% acetonitrile washes. Recovered peptides were dried in SpeedVac and reconstituted in 10 μL of 2% acetonitrile 0.1% TFA for LC-MALDI analysis.

### LC-MALDI Analysis

Serum tryptic digest with or without deglycosylation was separated using DiNa nano-HPLC system (KYA Technologies, Tokyo, Japan). Solvent A was 2% acetonitrile and 0.1% TFA in water and solvent B was 70% acetonitrile and 0.1% TFA in water. 2 μL sample, a final amount equivalent to 0.4 μL serum, was injected onto a trap column (L-column ODS, 5 μm, 0.3 × 5 mm, CERI, Saitama, Japan) and loaded by 8 μL/min flow of solvent A. At 5 min, valve was switched and the peptides were separated by an in-house packed analytical column (L-column ODS, 3 μm, in 0.1 mm × 200 mm capillary) at 200 nL/min flow rate using the following gradient: 5 min, 2% solvent B; 6 min, 10% solvent B; 90 min, 55% solvent B; 95 min, 100% solvent B; and 110 min, 100% solvent B. The column end was connected directly to the spotting tip of DiNA MAP target plate spotting device (KYA Technologies). CHCA matrix solution was prepared at 1.5 mg/mL concentration in 70% acetonitrile, 0.1% TFA and 0.03 mg/mL ammonium citrate, which was pumped to the spotting tip at 2.2 μL/min flow rate and therein mixed with column elution. The mixture was deposited onto a 1536-well μFocusing plate (Hudson Surface Technologies Inc., Newark, NJ) every 15 seconds between 20.0 to 109.75 minutes for a total of 360 spot fractions. Mass spectrometric analysis was performed using 4800 Plus MALDI TOF/TOF Analyzer (AB Sciex, Foster City, CA) operated on 4000 Series Explorer software version 3.5. For each fraction spot, data was accumulated from 1000 laser shots in a randomized raster of 400 μm diameter over mass range m/z 800-4000. The laser repeat rate was 200 Hz and the laser power was fixed at 3500 units throughout the experiment. 5 calibration spots comprising 6 standard peptides were used for external calibration.

### Data analysis

Individual MALDI MS raw data was exported as t2d file, ordered in chromatographic order and imported into Expressionist Refiner MS system (Genedata AG, Basel, Switzerland), where they were combined and displayed as mass chromatograms. Default processing parameters were applied unless otherwise specified. The chromatogram data was first simplified by subtracting the background noise by using the following criteria: 0.3 min RT window, 40% quantile subtraction, 0.15 point RT smoothing. After subtraction, all data points below threshold intensity of "100" were clipped to zero. A set of chromatograms were then aligned in the RT direction by nonlinear transformation, mapping the original time onto a common universal retention time, to ensure that equal RT values correspond to the elution of the same compounds. The following parameters were applied: RT transformation window, 5 min; RT search interval, 30 min; m/z window, 0.2 Da; gap penalty, 1. Peak signals were detected by summed peak detection algorithm, which computes a temporary averaged chromatogram over all input chromatograms, thereby allowing them to share the matching set of peaks with identical boundaries. Here, the summation windows of 0.2 Da in the m/z direction and 1 minute in the RT direction were selected. The detected peaks were grouped into isotopic clusters of individual compounds by summed isotope clustering activity, using the following parameters: minimum charge, 1; maximum charge, 2; maximum missing peaks, 1; first allowed gap position, 3; RT window, 1 min; peptide isotope shaping tolerance, 0.8.

### Statistical analysis

The cluster information generated by Refiner MS was imported into Genedata Expressionist Analyst software for statistical analysis. The clusters were first filtered by a valid value proportion of 100% (i.e. signal was detected in all of the experimental replicates). All of these clusters were subjected to t-test for extracting differentially expressed clusters between the experimental groups, where P < 0.01 was considered to be significant. Present/absent search was performed to select for clusters with 0 or 1 counterpart detection, which were omitted by t-test.

### Protein identification

As an exhaustive study, full MS/MS analysis was performed on three of the replicate runs. Precursor peaks were selected according to the software interpretation algorithm, while limiting the maximum number of acquisition to 10 per spot. Precursor peaks were measured in descending order of intensity. Precursor ions were isolated at 150 FWHM resolution, fragmentation was induced without the use of collision gas at 6 kV and fragment ions were further accelerated at 15 kV. Laser power of 4200 units was used, and the acquisition was summed over 2000 laser shots or until 4 fragment peaks exceeded S/N 100. Protein Pilot software version 2.0 was used to generate MS/MS peak lists for searching by MASCOT[32] version 2.2.03 (Matrix Science, London, U.K.) against 20345 human sequences of SwissProt version 57.14. Prior to search, a custom +3 Da modification on asparagine residue resulting from deglycosylation in H_2_^18^O was defined. The search parameters were as follows: enzyme, trypsin (allow up to 2 missed cleavages); fixed modification, carbamidomethyl; variable modifications, ^18^O-deglycosylation (Asn); peptide tolerance, 300 ppm; MSMS tolerance, 0.5 Da. Ion expectation score of 0.05 was used for the cut-off line for identification. For candidate biomarker peptides that were not identified by this method, searching was iteratively repeated in different search parameters, such as "semitrypsin" enzyme restriction, "N-terminal pyroglutamic acid" and "+365 Da modification on Thr" (corresponding to O-linked N-acetylhexosamine and hexose attached to a threonine residue) as variable modifications.

### Multiple reaction monitoring

2 μL of the serum tryptic digest analyzed by LC-MALDI was diluted by adding 6 μL of solvent A and 4 μL of tBSA proteomic standard (KYA Technologies) dissolved at 50 fmol/μL. 1 μL of this mixture was injected for a single analysis. Paradigm nano-HPLC system with PAL autoinjector was used for separation. Solvent A was 2% ACN in 0.1% formic acid, solvent B was 90% ACN in 0.1% formic acid, and sample was loaded with 2% ACN in 0.1% TFA. The trap column was L-column ODS, 5 μm, 0.3 × 5 mm, and the analytical column was L-column ODS loaded in-house directly into a sprayer tip (GL Science, Tokyo, Japan). MRM was performed using 4000QTRAP mass spectrometer (AB Sciex) during a 13 minutes gradient (2-55% solvent B) at 200 nL/min flow rate. 70 ions were monitored simultaneously for 30 minutes, each transition with 20 ms dwell time with 5 ms interval, taking a total of 1.75 s per scan. Transitions were selected from series of pilot experiment in which in-silico developed transitions for each peptide were tested for signal intensity and specificity. Transitions were considered as the derivative of target peptide only when all of them responded simultaneously and the retention time of detection matched that of LC-MALDI data. Instrument settings were as follows: declustering potential, 70; entrance potential, 10; curtain gas, 10; collision gas, 4; ion spray voltage, 2100; ion source gas, 10; interphase heater temperature, 150°C. Peak areas were integrated using MultiQuant software version 1.1.0.26. Raw data was normalized to the total signal acquired, which includes two spiked-in BSA fragments detected at highest intensity.

### Immunoblot analysis

For the verification study, crude serum samples from fresh aliquots for all screening sample set (except for 3 normal controls N-3, 4, and 10, which were substituted by N-11, 12, and 13, respectively) were analyzed. 0.5 μL of crude serum was diluted 100-fold with SDS-PAGE sample buffer, boiled and 20 μL was used for immunoblot analysis. SDS-PAGE was performed using NuPAGE Bis-Tris 4-12% acrylamide gel with 2-morpholinoethanesulfonic acid buffer system, and electroblotted onto a PVDF membrane. The blots were probed with anti-C3 polyclonal antibody (Sigma, product code GW20073F) diluted 5000-fold in 5% skim milk, followed by incubation with horseradish peroxidise-conjugated secondary antibody (Sigma, product code A9046) diluted 10000-fold in 2% BSA. The reactivity was visualized on X-ray films using ECL detection kit (GE Healthcare). Immunoblot was also performed with anti-C3d monoclonal antibody (Abbiotec, San Diego, CA) and horseradish peroxidise-conjugated secondary antibody (GE Healthcare) to confirm specificity of the polyclonal antibody (Figure S-2).

## Competing interests

This study is supported in part by the grants from Toppan Printing Co., Ltd., Tokyo, Japan, and Shimadzu Corporation, Kyoto, Japan.

## Authors' contributions

AT performed all experiments and drafted the manuscript. HN and KM added statistical interpretation. NI and NK provided the serum samples and revised the manuscript. YD and TAS contributed to mass spectrometry analysis. YN critically revised the manuscript. KU conceived the study, helped in experimental design and critically revised the manuscript. All authors read and approved the final manuscript.

## Supplementary Material

Additional file 1 **Supplementary Information**. This PDF file contains the following material: Figure S-1, a histogram and summary of the number of reproducible peaks. Figure S-2, an immunoblot showing the specificity of antibodies used. Table S-1, the list of serum samples. Table S-2, the list of glycopeptides identified in this study. Table S-3, the detail of MRM transitions used for verification analysis. Click here for file
