# Supplementary material for: Deglycosylation and label-free quantitative LC-MALDI MS applied to efficient serum biomarker discovery of lung cancer
Source: Proteome Sci. 2011 Apr 8;9:18. doi: 10.1186/1477-5956-9-18 (PMC3090313; doi:10.1186/1477-5956-9-18)
Supplement: Additional file 1 — Supplementary Information. This PDF file contains the following material: Figure S-1, a histogram and summary of the number of reproducible peaks. Figure S-2, an immunoblot showing the specificity of antibodies used. Table S-1, the list of serum samples. Table S-2, the list of glycopeptides identified in this study. Table S-3, the detail of MRM transitions used for verification analysis. [file 1477-5956-9-18-S1.PDF]

Supplementary Information for

Deglycosylation and label-free quantitative LC-MALDI MS applied to efficient serum biomarker discovery of lung cancer

Atsuhiko Toyama[1, 2], Hidewaki Nakagawa[2], Koichi Matsuda[1, 3], Nobuhisa Ishikawa[4], Nobuoki Kohno[4], Yataro Daigo[1, 3], Taka-Aki Sato[5], Yusuke Nakamura[3], and Koji Ueda[2]

This SI contains the following:

Figure S-1	A histogram and summary of the number of reproducible peaks
Figure S-2	A western blot analysis showing the specificity of antibodies used
Table S-1	The list of serum samples
Table S-2	The list of glycopeptides identified in this study
Table S-3	The detail of MRM transitions used for verification analysis

Supplementary Figure 1

A

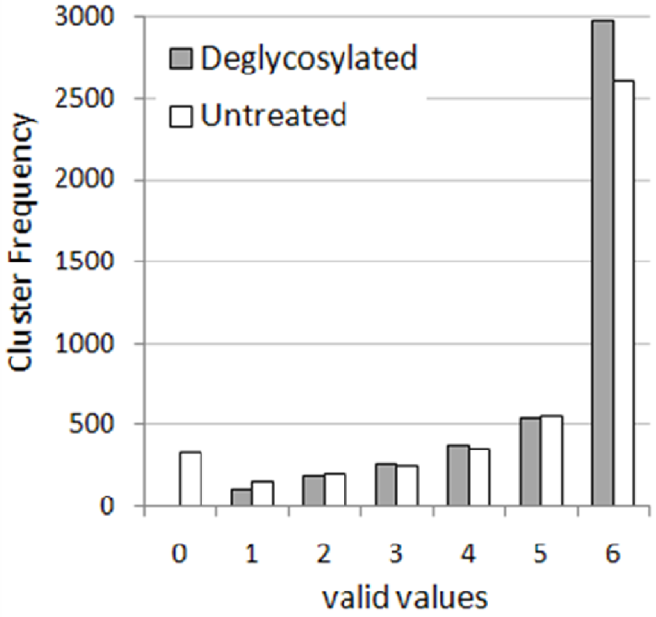

B

Valid Values in "Deglycosylated" (n=6)	Valid values in "Untreated" (n=6)						
	0	1	2	3	4	5	6
0	-	-	-	-	-	-	-
1	61	21	10	4	5	1	-
2	34	37	44	21	33	7	6
3	39	25	37	54	55	39	15
4	30	26	51	62	64	79	60
5	38	14	32	55	87	141	173
6	130	27	27	48	110	286	2356

Figure S-1. Valid value (number of non-zero data in the experimental group) as a measure of data quality. (**A**) valid value histogram plotting the frequency of clusters containing the given valid value(s): filled bars, deglycosylated group (n = 6), blank bars, untreated group (n = 6). (**B**), summary showing the number of clusters corresponding to the given combination of valid values. 2,356 clusters have valid value of 6 in both experimental groups, i.e. expression was observed in all samples in the analysis.

Supplementary Figure 2

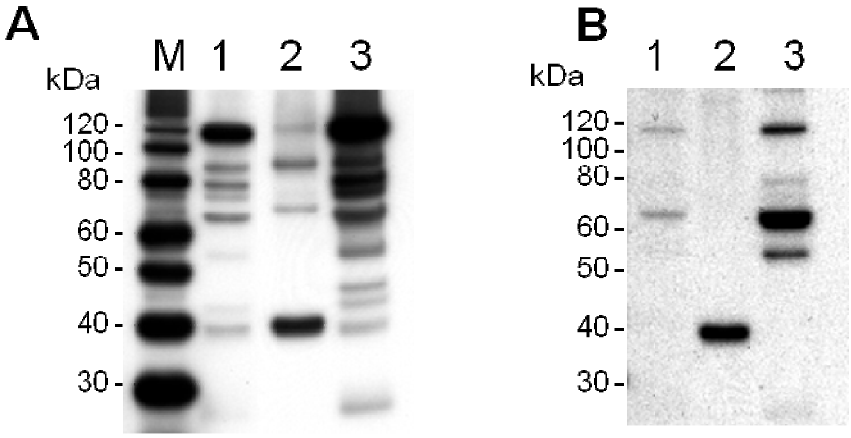

Figure S-2. Western blot analysis showing the specificity of the antibodies used: (**A**) chicken polyclonal to recombinant full-length C3; (**B**) mouse monoclonal antibody raised against C3d fragment. Lanes: *M*, molecular weight marker with cross-reactivity with the secondary antibody; *Lane 1*, 0.01 μL crude serum of healthy volunteer; *Lane 2*, flow-through fraction of MARS Hu-14 depletion; *Lane 3*, bound fraction of MARS Hu-14 depletion.

Table S-1. List of serum samples used in this study.

ID	Clinical Status	Age	Gender
N1	Healthy control	57	M
N2	Healthy control	56	M
N3	Healthy control	58	M
N4	Healthy control	58	M
N5	Healthy control	59	M
N6	Healthy control	58	M
N7	Healthy control	58	M
N8	Healthy control	60	M
N9	Healthy control	59	M
N10	Healthy control	57	M
N11	Healthy control	60	M
N12	Healthy control	57	M
N13	Healthy control	56	M
I-1	Lung adenocarcinoma, Stage-I	74	M
I-2	Lung adenocarcinoma, Stage-I	66	M
I-3	Lung adenocarcinoma, Stage-I	70	M
I-4	Lung adenocarcinoma, Stage-I	63	M
I-5	Lung adenocarcinoma, Stage-I	75	M
II-1	Lung adenocarcinoma, Stage-II	57	M
II-2	Lung adenocarcinoma, Stage-II	72	M
II-3	Lung adenocarcinoma, Stage-II	45	M
II-4	Lung adenocarcinoma, Stage-II	66	M
II-5	Lung adenocarcinoma, Stage-II	46	M
III-1	Lung adenocarcinoma, Stage-IIIb	76	M
III-2	Lung adenocarcinoma, Stage-IIIb	60	M
III-3	Lung adenocarcinoma, Stage-IIIb	42	M
III-4	Lung adenocarcinoma, Stage-IIIb	67	M
III-5	Lung adenocarcinoma, Stage-IIIb	79	M
IV-1	Lung adenocarcinoma, Stage-IV	58	M
IV-2	Lung adenocarcinoma, Stage-IV	72	M
IV-3	Lung adenocarcinoma, Stage-IV	83	M
IV-4	Lung adenocarcinoma, Stage-IV	55	M
IV-5	Lung adenocarcinoma, Stage-IV	61	M

Table S-2. List of 153 originally-glycosylated peptides that were identified with ^{18}O-incorporated N-glycosylation sites.

Accession	Protein name	MOWSE score	Exp. Score	Peptide sequence	Next residue
AFAM_HUMAN	Afamin	118	1.0E-10	NCCNTENPPGCYR	Y
		100	7.4E-09	YAEDKFNETTEK	S
		96	2.3E-08	ICAMEGLPQKHNFSHCCSK	V
		93	3.8E-08	YAEDKFNETTEKSLK	M
		75	1.7E-06	ICAMEGLPQKHNFSHCCSKVDAQR	R
		60	7.8E-05	DIENFNSTQK	F
		57	1.3E-04	DLLRNCCNTENPPGCYR	Y
		53	3.9E-04	HNFSHCCSKVDAQR	R
		49	9.1E-04	HNFSHCCSK	V
		43	0.005	IVQIYKDLLRNCCNTENPPGCYR	Y
		39	0.009	NCCNTENPPGCYR	Y
AACT_HUMAN	Alpha-1-antichymotrypsin	213	3.4E-20	TLNQSSDELQLSMGNAMFVK	E
		152	3.8E-14	GLKFNLTETSEAEIHQSFQHLLR	T
		119	8.4E-11	FNLTETSEAEIHQSFQHLLR	T
		92	4.5E-08	YTGNASALFILPDQDKMEEVEAMLLPETLKR	W
		84	2.8E-07	KLINDYVKNGTR	G
		71	5.4E-06	LINDYVKNGTR	G
A1BG_HUMAN	Alpha-1B-glycoprotein	197	2.1E-18	EGDHEFLEVPEAQEDVEATFPVHQPGNYSCSYR	T
		72	4.5E-06	LHDNQNGWSGDSAPVELILSDETLPAPEFSPEPESGR	A
		61	7.7E-05	LETPDFQLFKNGVAQEPVHLDSPAIK	H
		39	0.010	REGDHEFLEVPEAQEDVEATFPVHQPGNYSCSYR	T
FETUA_HUMAN	Alpha-2-HS-glycoprotein	214	2.6E-20	AALAAFNAQNNGSNFQLEEISR	A
		124	2.4E-11	VCQDCPLLAPLNDTRVVHAAK	A
		99	8.5E-09	VCQDCPLLAPLNDTR	V
		69	8.7E-06	KVCQDCPLLAPLNDTR	V
		59	7.3E-05	KVCQDCPLLAPLNDTRVVHAAK	A
		46	0.002	CNLLAEKQYGFCK	A
ANT3_HUMAN	Antithrombin-III	161	4.5E-15	SLTFNETYQDISELVYGAK	L
		135	2.0E-12	LGACNDTLQQLMEVFKFDTISEK	T
		76	1.7E-06	WVSNKTEGR	I
		74	2.8E-06	AAINKWVSNKTEGR	I
		64	2.4E-05	LFGDKSLTFNETYQDISELVYGAK	L
APOB_HUMAN	Apolipoprotein B-100	107	1.5E-09	QVFPGLNYCTSGAYSNASSTDSASYYPLTGDTR	L
		107	1.6E-09	QVFPGLNYCTSGAYSNASSTDSASYYPLTGDTR	L
		52	4.9E-04	FEVDSPVYNATWSASLK	N
APOH_HUMAN	Beta-2-glycoprotein 1	72	4.1E-06	VYKPSAGNNSLYR	D
		49	8.3E-04	LGNWSAMPSCK	A
C4BPA_HUMAN	C4b-binding protein alpha chain	50	0.001	LSVDKDQYVEPENVTIQCDSGYGVVGPQSITCSGNR	T
		43	0.003	TLFCNASKEWDNTTTECR	L
CERU_HUMAN	Ceruloplasmin	167	1.6E-15	EHEGAIYPDNTTDFQR	A
		156	1.5E-14	ENLTAPGSDSAVFFEQGTTR	I
		151	5.7E-14	AGLQAFFQVQECNKSSSK	D
		137	1.1E-12	AGLQAFFQVQECNKSSSKDNIR	G
		114	2.4E-10	FNKNNEGTYYSPNYNPQSR	S
		98	9.1E-09	ELHHLQEQNVSNAFLDKGEFYIGSK	Y
		63	3.2E-05	AGLQAFFQVQECNK	S
		62	4.3E-05	NNEGTYYSPNYNPQSR	S
		44	0.002	MYSVNGYTFGSLPGLSMCAEDRVK	W
CLUS_HUMAN	Clusterin	104	2.6E-09	LANLTQGEDQYYLR	V
		77	1.9E-06	LKELPGVCNETMMALWEECKPCLK	Q

		53	2.8E-04	ELPGVCNETMMALWEECKPCLK	Q
		51	5.1E-04	MLNTSSLLEQLNEQFNWVSR	L
		47	0.002	YVNKEIQNAVNGVK	Q
		45	0.002	EIRHNSTGCLR	M
FA12_HUMAN	Coagulation factor XII	59	9.5E-05	RNHSCEPCQTLAVR	S
		59	1.2E-04	TTLSGAPCQPWASEATYRNVTAEQAR	N
		57	1.3E-04	NHSCEPCQTLAVR	S
C1QA_HUMAN	Complement C1q subcomponent subunit A	66	2.6E-05	NPPMGGNVVIFDTVITNQEEPYQNHSGR	F
		42	0.006	RNPPMGGNVVIFDTVITNQEEPYQNHSGR	F
C1S_HUMAN	Complement C1s subcomponent	41	0.006	NCGVNCSGDVFTALIGEIASPNYPKPYPENSR	C
CO2_HUMAN	Complement C2	92	4.6E-08	LGSYPVGGNVSFECEDGFILR	G
CO4A_HUMAN	Complement C4-A	130	7.2E-12	FSDGLESNSSTQFEVK	K
		111	5.3E-10	NGFKSHALQLNNR	Q
		108	1.1E-09	FSDGLESNSSTQFEVKK	Y
		40	0.006	TLEIPGNSDPNMIPDGDFNSYVR	V
		35	0.019	QYRNGESVK	L
		34	0.027	GLNVTLSSTGR	N
CO5_HUMAN	Complement C5	70	8.4E-06	ANISHKDMQLGR	L
CO6_HUMAN	Complement component C6	67	1.7E-05	KLECNGENDCGDNSDERDCGR	T
CO7_HUMAN	Complement component C7	42	0.004	NYTLTGR	D
		43	0.005	SLVCNGDSDCDEDSADEDRCEDSER	R
CO8A_HUMAN	Complement component C8 alpha chain	74	2.7E-06	GGSSGWSGGLAQNR	S
CO8B_HUMAN	Complement component C8 beta chain	88	1.7E-07	LLCNGDNDCGDQSDEANCRR	I
CO9_HUMAN	Complement component C9	183	3.3E-17	AVNITSENLIDDVVSLIR	G
CFAB_HUMAN	Complement factor B	156	1.7E-14	SPYYNVSDEISFHCYDGYTLR	G
		63	4.8E-05	WSGQTAICDNGAGYCSNPGIPIGTRK	V
		55	3.7E-04	ALQAVYSMMSWPDDVPPEGWNR	T
		53	4.8E-04	WSGQTAICDNGAGYCSNPGIPIGTR	K
		45	0.003	KALQAVYSMMSWPDDVPPEGWNR	T
		38	0.010	ALQAVYSMMSWPDDVPPEGWNRTR	H
		35	0.025	GSANRTCQVNGR	W
CFAH_HUMAN	Complement factor H	157	1.2E-14	IPCSQPPQIEHGTINSSR	S
		103	3.2E-09	ISEENETTCYMGK	W
		100	8.0E-09	WQSIPLCVEKIPCSQPPQIEHGTINSSR	S
		72	4.6E-06	TGDEITYQCRNGFYPATR	G
		49	7.9E-04	CYFPYLENGYNQNYGR	K
		44	0.003	ITCRNGQWSEPPK	C
		32	0.043	MDGASNVTCINSR	W
FHR1_HUMAN	Complement factor H-related protein 1	100	7.4E-09	LQNNENNISCVER	G
CFAI_HUMAN	Complement factor I	77	1.6E-06	NGTAVCATNRR	S
		42	0.004	FLNNGTCTAEGK	F
		42	0.006	SIPACVPWSPYLFQPNDTCIVSGWGR	E
DCAF6_HUMAN	DDB1- and CUL4-associated factor 6	31	0.040	ATGNYAGR	G
FETUB_HUMAN	Fetuin-B	39	0.009	GCNDSDVLAVAGFALR	D
FINC_HUMAN	Fibronectin	95	2.7E-08	HEEGHMLNCTCFGQGR	G
		59	8.3E-05	DQCIVDDITYNVNDTFHKR	H
		51	4.6E-04	RHEEGHMLNCTCFGQGR	G
HEMO_HUMAN	Hemopexin	136	2.0E-12	NGTGHGNSTHHGPEYMR	C
		111	5.5E-10	SWPAVGNCSSALR	W
		109	9.2E-10	ALPQPQNVTSLLGCTH	-
		88	1.8E-07	GHGHRNGTGHGNSTHHGPEYMR	C
		47	0.001	ERSWPAVGNCSSALR	W
		46	0.003	GHGHRNGTGHGNSTHHGPEYMR	C
		40	0.008	SLGPNSCSANGPGLYLIHGPNLYCYSDVEK	L
HGFL_HUMAN	Hepatocyte growth factor-like protein	66	2.5E-05	AFHYNVSSHGCQLLPWTQHSPHTR	L
		37	0.015	GKGEGYRGTANTTTAGVPCQR	W

HRG_HUMAN	Histidine-rich glycoprotein	41	0.008	VIDFNCTTSSVSSALANTKDSPVLIDFFEDTER	Y
ALS_HUMAN	Insulin-like growth factor-binding protein complex acid labile subunit	98	1.1E-08	AGAFLGLTNVAVMNLSGNCLR	N
ITIH1_HUMAN	Inter-alpha-trypsin inhibitor heavy chain H1	54	2.1E-04	ANLSSQALQMSLDYGFVTPLTSMSIR	G
ITIH2_HUMAN	Inter-alpha-trypsin inhibitor heavy chain H2	78	1.2E-06	GAFISNFSMTVDGK	T
		62	4.3E-05	GAFISNFSMTVDGKTFR	S
		57	1.3E-04	LSNENHGIAQR	I
ITIH3_HUMAN	Inter-alpha-trypsin inhibitor heavy chain H3	47	0.002	NAHGEEKENLTAR	A
ITIH4_HUMAN	Inter-alpha-trypsin inhibitor heavy chain H4	153	3.3E-14	LPTQNITFQTESSVAEQEAEFQSPK	Y
		33	0.043	VQGNDHSATRER	R
KI13B_HUMAN	Kinesin-like protein KIF13B	33	0.039	HIVNNAVVNEDPNARIIR	D
KNG1_HUMAN	Kininogen-1	179	7.7E-17	HGIQYFNNNTQHSSLFMLNEVKR	A
		122	4.6E-11	YNSQNQSNNQFVLYR	I
		121	7.5E-11	HGIQYFNNNTQHSSLFMLNEVK	R
		110	6.0E-10	YNSQNQSNNQFVLYR	I
		80	7.0E-07	LNAENNATFYFK	I
		45	0.002	ITYSIVQTNCSKENFLFLTPDCK	S
A2GL_HUMAN	Leucine-rich alpha-2-glycoprotein	108	1.1E-09	KLPPGLLANFTLLR	T
		34	0.029	LPPGLLANFTLLR	T
LUM_HUMAN	Lumican	68	9.9E-06	LGSFEGLVNLTFIHLQHNR	L
PGRP2_HUMAN	N-acetylmuramoyl-L-alanine amidase	119	7.7E-11	GFGVAIVGNYTAALPTEAALR	T
KLKB1_HUMAN	Plasma kallikrein	91	5.2E-08	IYPGVDFGGEELNVTFVK	G
		87	1.1E-07	GVNFNVSKVSSVEECQKR	C
IC1_HUMAN	Plasma protease C1 inhibitor	69	8.0E-06	VLSNNSDANLELINTWVAK	N
		48	0.001	VLSNNSDANLELINTWVAK	N
		40	0.007	GVTSVSQIFHSPDLAIRDTFVNASR	T
		38	0.013	VGQLQLSHNLSLVILVPQNLK	H
IPSP_HUMAN	Plasma serine protease inhibitor	49	8.3E-04	VVGVPYQGNATALFILPSEGK	M
PLMN_HUMAN	Plasminogen	38	0.011	TKNGITCQK	W
		36	0.016	VYLSECKTGNGKNYR	G
		35	0.021	TGNGKNYR	G
AMBP_HUMAN	Protein AMBP	57	1.6E-04	CVLFPYGGCQGNGNKFYSEKECR	E
SAMP_HUMAN	Serum amyloid P-component	142	5.7E-13	ESVTDHVNLITPLEKPLQNFTLCFR	A
TSP1_HUMAN	Thrombospondin-1	34	0.024	VSCPIMPCSNATVPDGECCPR	C
BDP1_HUMAN	Transcription factor TFIIIB component B homolog	35	0.007	GASNTQLLLK	E
		38	0.014	KFKPNVTR	G
GCN1L_HUMAN	Translational activator GCN1	36	0.018	LDAGNQLALIEELHK	E
		35	0.029	NGLNLLR	R
K1377_HUMAN	Uncharacterized protein KIAA1377	43	0.003	NTLQIIPLLEKR	E
VTDB_HUMAN	Vitamin D-binding protein	76	1.9E-06	LCDNLSTKNSKFEDCCQEK	T
		60	6.0E-05	NSKFEDCCQEK	T
		37	0.018	HQPQEFPTYVEPTNDEICEAFRKDPK	E
		34	0.037	RSDFASNCCSINSPPLYCDSEIDAELKNIL	-
VTNC_HUMAN	Vitronectin	188	1.8E-17	NISDGFDGIPDNVDAALALPAHSYSGR	E
		43	0.004	NGSLFAFR	G
ZN680_HUMAN	Zinc finger protein 680	36	0.017	AFNWFATLTNHKR	I
ZA2G_HUMAN	Zinc-alpha-2-glycoprotein	32	0.045	FGCEIENNR	S
		61	4.8E-05	FGCEIENNRSSGAFWK	Y

Table S-3. List of MRM transitions used for verification of MALDI MS quantification

Protein Name	Transition ID	Peptide sequence	Q1 mass	Q3 mass	Dwell Time (ms)	Collision Energy (V)
Alpha-1B-glycoprotein	A1BG__FALVR__+2__b7	FALVREDR	503.27	831.4	20	28.1
Alpha-1-antichymotrypsin	AACT__EQLSL__+3__y10	EQLSLLDRFTEDAK	555.61	604.3	20	20.0
Bovine serum albumin	BSA_1	AEFVEVTK	461.8	722.4	20	29.1
Bovine serum albumin	BSA_7	LVNELTEFAK	582.3	951.5	20	34.4
Complement C1r subcomponent	C1R__DYFIA__+2__y5	DYFIATCK	509.23	592.3	20	28.4
Ceruloplasmin	CERU__YTVNQ__+2__y3	YTVNQCR	470.71	463.2	20	26.7
Clusterin	CLUS__YVNKE__+2__y4	YVNKEIQNAVNGVK	788.92	418.2	20	40.7
Complement C2	CO2__TAVDH__+2__b6	TAVDHIREILNINQK	882.49	637.3	20	44.8
Complement C2	CO2__TAVDH__+2__y8	TAVDHIREILNINQK	882.49	971.6	20	44.8
Complement C3	CO3__AGDFL__+2__y9	AGDFLEANYMNLQR	821.38	1138.5	20	42.1
Complement C3	CO3__ILLQG__+3__b6	ILLQGTPVAQMTEDAVDAER	719.69	626.4	20	24.7
Complement C3	CO3__KGYTQ__+2__y5	KGYTQQLAFR	606.325	634.4	20	32.7
Complement C3	CO3__QPSSA__+2__b9	QPSSAFAAFVKR	654.85	907.4	20	34.8
Complement C3	CO3__WLNEQ__+2__y5	WLNEQR	423.21	659.3	20	24.6
Complement Component C9	CO9__FTPTE__+4__y15	FTPTETNKAEQCCEETASSISLHGK	707.07	853.9	20	23.2
Complement Component C9	CO9__QYTTS__+3__pep	QYTTSYDPELTESSGSASHIDCR	984.74	1294.1	20	32.4
Carboxypeptidase N subunit 2	CPN2__SQCTY__+3__y7	SQCTYSNPEGTVVLACDQAQCR	848.69	937.4	20	28.5
Carboxypeptidase N subunit 2	CPN2__SQCTY__+3__y8	SQCTYSNPEGTVVLACDQAQCR	848.69	1008.4	20	28.5
Fetuin-B	FETUB__MSPPQ__+2__y7	MSPPQLALNPSALLSR	847.96	743.4	20	43.31
Fibronectin	FINC__AQITG__+2__y2	AQITGYR	404.71	395.2	20	23.8
Fibronectin	FINC__AQITG__+2__y3	AQITGYR	404.71	496.3	20	23.8
Fibronectin	FINC__GFNCE__+4__y17	GFNCESKPEAEETCFDKYTGNTYR	726.56	1040.9	20	23.8
Hepatocyte growth factor-like protein	HGFL__RVDRL__+2__y5	RVDRLDQR	529.29	687.4	20	29.3
Inter-alpha-trypsin inhibitor heavy chain H3	ITIH3__EHLVQ__+2__b3	EHLVQATPENLQEAR	867.94	380.2	20	44.2
Inter-alpha-trypsin inhibitor heavy chain H4	ITIH4__EKNGI__+3__y5	EKNGIDIYSLTVDSR	570.95	577.3	20	20.4
Inter-alpha-trypsin inhibitor heavy chain H4	ITIH4__ETLFS__+2__y8	ETLFSVMPGLK	611.33	878.5	20	32.9
Inter-alpha-trypsin inhibitor heavy chain H4	ITIH4__MNFRP__+3__y9+	MNFRPGVLSSR	421.89	509.8	20	10.0
Kininogen-1	KNG1__RPPGF__+2__y2	RPPGFSPF	452.73	263.1	20	25.9
